# Targeting Splicing Factor SRSF6 for Cancer Therapy

**DOI:** 10.3389/fcell.2021.780023

**Published:** 2021-11-30

**Authors:** Wenting She, Jun Shao, Rong Jia

**Affiliations:** ^1^ The State Key Laboratory Breeding Base of Basic Science of Stomatology (Hubei-MOST) & Key Laboratory of Oral Biomedicine Ministry of Education, School & Hospital of Stomatology, Wuhan University, Wuhan, China; ^2^ Department of Stomatology, Renmin Hospital of Wuhan University, Wuhan University, Wuhan, China; ^3^ Department of Breast Surgery, Hubei Cancer Hospital, Tongji Medical College, Huazhong University of Science and Technology and Hubei Provincial Clinical Research Center for Breast Cancer, Wuhan, China

**Keywords:** SRSF6, oncogene, alternative splicing, cancer therapy, overexpression

## Abstract

Aberrant alternative splicing of pre-mRNA is an emerging cancer hallmark. Many cancer-associated genes undergo alternative splicing to produce multiple isoforms with diverse or even antagonistic functions. Oncogenic isoforms are often up-regulated, whereas tumor suppressive isoforms are down-regulated during tumorigenesis. Serine/arginine-rich splicing factor 6 (SRSF6) is an important splicing factor that regulates the alternative splicing of hundreds of target genes, including many cancer-associated genes. The potential roles of SRSF6 in cancers have attracted increasing attentions in the past decade. Accumulated pieces of evidence have shown that SRSF6 is a potential oncogenic gene that promotes oncogenic splicing when overexpressed. Targeting SRSF6 may suppress tumorigenesis. In this review, we describe the gene, mRNA, and protein structure of SRSF6; summarize the current understanding of the expression, functions, and regulatory mechanisms of SRSF6 during tumorigenesis; and discuss the potential application of targeting SRSF6 in cancer treatment.

## 1 Introduction

Serine/arginine-rich splicing factor 6 (SRSF6), also called SRp55 or SFRS6, was initially identified in Drosophila by using mAb104, a monoclonal antibody that recognizes phosphorylated serine/arginine-rich (SR) RNA-binding proteins (Roth et al., 1991). Human SRSF6 protein was also identified by the same antibody (Zahler et al., 1992), and its gene was cloned later (Screaton et al., 1995). SRSF6 is highly conserved across species and is the key regulator of RNA constitutive and alternative splicing. In Drosophila, SRSF6 plays important roles in tissue development (Fic et al., 2007), and the deletion of SRSF6 causes lethal defects during development (Ring and Lis, 1994). SRSF6 is a multi-function protein that is involved in several biological processes besides RNA splicing, including translation (Swanson et al., 2010) and transcription (Juge et al., 2010). Besides cancer, SRSF6 has been associated with numerous human diseases, such as pleural fibrosis (Liang et al., 2021), Huntington’s disease (Cabrera and Lucas, 2017), Alzheimer’s disease (Mai et al., 2019), diabetes (Juan-Mateu et al., 2018), and systemic sclerosis (Manetti et al., 2011).

Most eukaryotic genes contain both exons and introns. After transcription, introns should be spliced out from pre-mRNA, and exons are connected to produce mature mRNA, which is crucial for gene expression. However, the definition of exon or intron in pre-mRNA is not always constant (De Conti et al., 2013). Some exons or introns could be spliced alternatively, which is called alternative splicing (Berget et al., 1977; Chow et al., 1977). One gene can produce multiple transcripts via alternative splicing, which increases the encoding capacity of genomes dramatically and plays important roles on the regulation of gene expression (Kornblihtt et al., 2013). Notably, alternative splicing profiles in cancer cells are significantly different from normal cells (Cherry and Lynch, 2020). With the progress of transcriptomic sequencing, aberrant alternative splicing has been increasingly recognized as an important cause of cancer (Liu and Rabadan, 2021).

Splicing factors refer to the important regulators in the alternative splicing of pre-mRNA. Serine and arginine-rich (SR) proteins are major splicing factor family (Manley and Tacke, 1996). SRSF6 belongs to the SR protein family, which all possess at least one N-terminal RNA recognition motif (RRM) domain and a C-terminal RS domain, and play important roles in RNA alternative splicing (Shepard and Hertel, 2009). Strikingly, accumulated pieces of evidence have demonstrated that most SR members are involved in tumorigenesis (Kedzierska and Piekielko-Witkowska, 2017). The potential roles of SRSF6 in cancers have also attracted increasing attentions in the past decade. In this review, we attempted to summarize the current understanding toward the expression, functions and regulatory mechanisms of SRSF6 during tumorigenesis, and discuss the potential application of targeting SRSF6 in cancer treatment.

## 2 Gene, mRNA and Protein Structure of Serine/Arginine-Rich Splicing Factor 6

**FIGURE 1:**
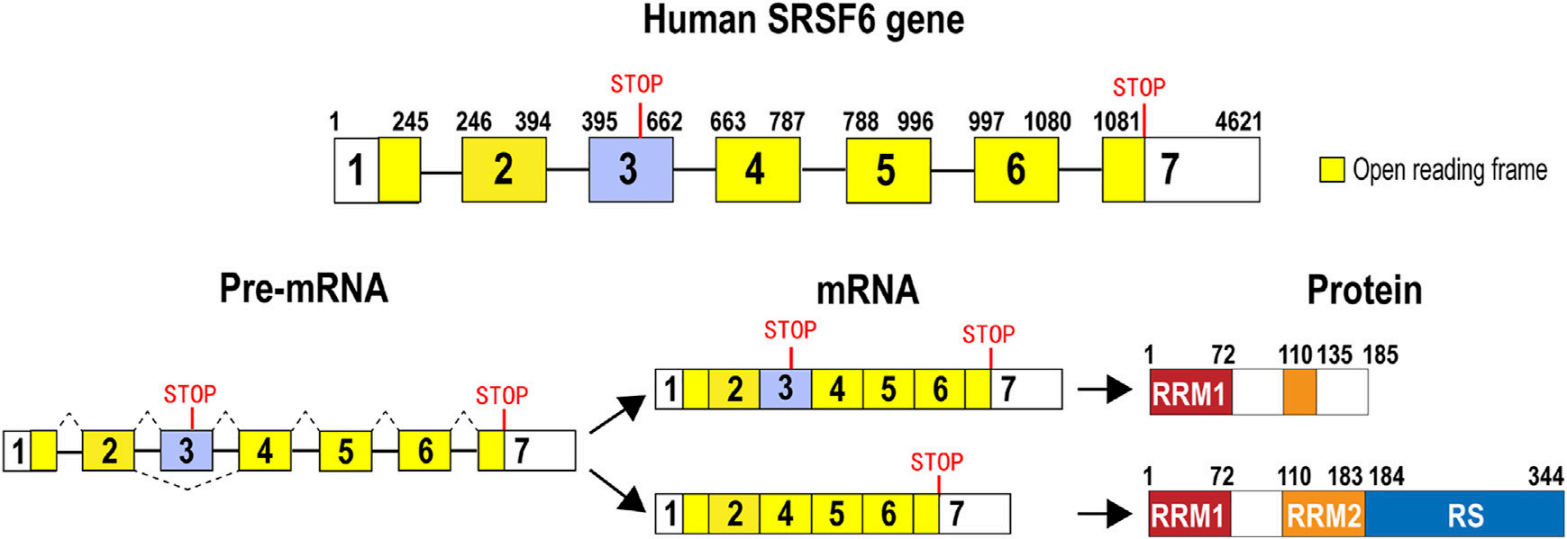
Gene, pre-mRNA, mature mRNA, and encoded protein structure of human SRSF6. Human SRSF6 gene contains 7 exons. Exon 3 is an alternative exon and contains an in-frame pre-mature stop codon. Transcripts with exon 3 may encode a truncated SRSF6. Transcripts without exon 3 encode full-length SRSF6 protein that has two RNA recognition domains (RRM1 or RRM2) and an arginine and serine-rich domain (RS).

Human SRSF6 gene is located in chromosome 20, and it includes at least seven exons and six introns. Exon 3 is an alternative exon that contains an in-frame stop codon. Therefore, isoform 2 with exon 3 (accession number: NR_034009) is a subject of nonsense-mediated decay (NMD). By contrast, isoform 1 without exon 3 (accession number: NM_006275) encodes a full-length SRSF6 protein, which has 344 amino acids, and includes a RRM 1 (RNA recognition motif 1), a RRM2 (also called RRM homolog, RRMH), and a C-terminal RS (arginine and serine dipeptides) domain (Zahler et al., 1992; Shepard and Hertel, 2009) that functions as a protein interaction domain. Isoform 2 may encode a truncated SRSF6 protein without most of RRM2 domain and whole RS domain (Figure 1). Similar to some other members of the SR protein family, SRSF6 is also a shuttle protein between the nucleus and the cytoplasm (Sapra et al., 2009), by which SRSF6 is also involved in translation.

## 3 The Normal Cellular Functions of Serine/Arginine-Rich Splicing Factor 6

SRSF6 plays important roles in normal cellular processes. For example, SRSF6 is required for the mitochondrial respiration process. SRSF6 knockdown decreased mitochondrial respiration and impaired ATP production and insulin release in human pancreatic β-cell (Juan-Mateu et al., 2018). Moreover, SRSF6 is negatively associated with cellular early responses to DNA damage (Filippov et al., 2007). Recently, Yang et al. showed that SRSF6 regulates the alternative splicing of a set of genes enriched in DNA damage response pathway including BRCA2 via transcriptomic analysis (Yang et al., 2020). Intriguingly, Tammaro et al. found that SRSF6 also controlled the inclusion of exon 11 of BRCA1, another gene responsible for DNA repair, by interacting with a splicing regulatory motif in exon 11 (Tammaro et al., 2014). SRSF6 may also be involved in the cell proliferation of some normal cells. Liang et al. showed that SRSF6 knockdown significantly inhibited cell proliferation of pleural mesothelial cells stimulated by inflammation (Liang et al., 2021). In neuron cells, SRSF6 helps maintain microtubule stability by promoting the inclusion of Tau exon 10, which is important for the assembly and stability of microtubule in neuron cells (Yin et al., 2012).

SRSF6 may also play roles in some other cellular processes. SRSF6 is a nucleocytoplasmic shuttling protein, which is similar to two other members of the SR protein family, namely, SRSF3 and SRSF7 (Sapra et al., 2009). SRSF3 can bind NXF1 and promote RNA export to cytoplasm (Huang and Steitz, 2001). SRSF7 can enhance the translation of constitutive transport element (CTD)-containing RNA (Swartz et al., 2007). Therefore, SRSF6 may also play roles in the export and translation of mRNA. Indeed, SRSF6 can enhance the translation of human immunodeficiency virus (HIV) type 1 Gag mRNA, which is an unspliced RNA (Swanson et al., 2010). Probably, SRSF6 may also regulate the translation of some cellular genes.

## 4 Expression and Clinical Significance of Serine/Arginine-Rich Splicing Factor 6 in Cancers

Several SR family members, such as SRSF1 (Karni et al., 2007), SRSF3 (Jia et al., 2010), and SRSF5 (Yang et al., 2018), have been demonstrated to be oncogenes and overexpressed in cancers. Recently, SRSF6 was also reported to be overexpressed in some cancers (Table 1).

**TABLE 1:** Expression and clinical significance of SRSF6 in cancers.

Cancer type	Methods	Expression of SRSF6 (cancer vs normal)	Clinical significance	References
Colorectal cancer	qRT-PCR	Up	Poor survival	Wan et al. (2019)
Colorectal cancer	Western blot	Up	None	Park et al. (2016)
Colorectal cancer	Western blot	Up	N/A	Si et al. (2021)
Colorectal cancer	Western blot	Down	N/A	Lin et al. (2017)
Colon cancer	RT-PCR	Up	N/A	Cohen-Eliav et al. (2013)
Leukemia	RNA-seq	N/A	Poor survival	Zhou et al. (2020b)
Lung cancer	RT-PCR	Up	N/A	Cohen-Eliav et al. (2013)
Skin cancer	Immunohistochemistry	Up	N/A	Jensen et al. (2014)
Ovarian cancer	RT-PCR	Up	N/A	Mayer et al. (2015)
Ovarian cancer	RT-PCR	No difference	N/A	Iborra et al. (2013)

### 4.1 Colon and Colorectal Cancer

SRSF6 expression in colon and colorectal cancer has been studied extensively. Most studies demonstrated that SRSF6 was overexpressed in cancer tissues. For example, Cohen-Eliav et al. reported that SRSF6 mRNA was overexpressed in colon cancer, and its gene was amplified in some colon cancer patients (37%) (Cohen-Eliav et al., 2013). Wan et al. showed that SRSF6 is significantly overexpressed in colorectal cancer patients from the TCGA database and their cohort in transcriptional level. Furthermore, SRSF6 overexpression is significantly associated with poor overall survival (Wan et al., 2019). Park et al. showed that SRSF6 protein was overexpressed in a cohort of colorectal patients by Western blot (Park et al., 2016), which was further confirmed in another study by Western blot (Si et al., 2021). However, Lin et al. showed that SRSF6 protein was downregulated in colorectal cancer tissues in eight patients compared with adjacent normal tissues by Western blot (Lin et al., 2017). More studies in larger cohorts may be required to evaluate SRSF6 expression in colorectal cancer.

### 4.2 Other Cancers

SRSF6 is significantly overexpressed in a set of subtypes of skin cancer, including basal-cell carcinoma, squamous-cell carcinoma, and malignant melanoma (Jensen et al., 2014). SRSF6 gene is also reported to be amplified in some lung cancer patients (12%) (Cohen-Eliav et al., 2013), and overexpressed (Cohen-Eliav et al., 2013; Kim et al., 2016).

In ovarian cancer, Mayer et al. showed that cancer tissues expressed significantly higher SRSF6 than normal tissues in a small cohort (Mayer et al., 2015). However, another study showed that the expression level of SRSF6 is not significantly superior to normal controls in a small cohort of patients with ovarian cancer. Interestingly, patients with metastasis showed significant higher SRSF6 expression (Iborra et al., 2013). More studies in larger cohorts are required to determine the association between SRSF6 expression and ovarian cancer. In addition, Li et al. showed that pancreatic cancer tissues expressed less SRSF6 than adjacent normal tissues, suggesting that SRSF6 may be not overexpressed in some cancers (Li et al., 2020).

So far, only a few studies reported the relationship between SRSF6 expression and cancer patient prognosis. Besides colorectal cancer, SRSF6 expression is also associated with poor prognosis in T-cell acute lymphoblastic leukemia (T-ALL) (Zhou et al., 2020b). More pieces of evidence are required to determine the association between SRSF6 expression and cancer patient prognoses.

In summary, SRSF6 may be overexpressed in most cancers. However, the association between SRSF6 expression and disease prognosis remains largely unclear. Therefore, to understand the value of SRSF6 expression in cancer diagnosis and prognosis, more studies are required to investigate the expression and clinical significance of SRSF6 in cancers.

## 5 Mutations of Serine/Arginine-Rich Splicing Factor 6 in Cancers

Gene mutation is an important cause of cancer. Mutations of some splicing factors have been reported in hematological malignancies and solid cancers (Yoshida and Ogawa, 2014). For example, splicing factor SF3B1 mutation led to the missplicing and downregulation of PPP2R5A gene and resulted in the stabilization of Myc protein and promotion of tumorigenesis (Liu et al., 2020). SRSF2 mutations altered its binding specificity from G-rich sequences to C-rich sequences (Kim et al., 2015) and associated with poor outcome in patients with leukemic transformation of myeloproliferative neoplasms (Zhang et al., 2012). Mutations in SRSF6 gene may also change its binding specificity. So far, only few studies reported mutations in SRSF6 gene. We summarized SRSF6 mutations in different types of cancer according to cBioPortal online TCGA cancer database (Table 2). SRSF6 mutation frequencies in cancers are relatively lower (<5%) compared with those in SRSF2 [10–50% in hematologic malignancies (Chen et al., 2021b)]. Further studies are required to understand the roles of SRSF6 mutations in cancer.

**TABLE 2:** Mutations of SRSF6 gene in cancers according to cBioPortal online TCGA cancer database.

Type of cancer	Alteration Frequency (%)					
	Missense_mutation	Nonsense_mutation	Frameshift mutation	In frame deletion	Splice site	Fusion gene
Colorectal adenocarcinoma	1.18	0.5	0.34	0	0	0.17
Stomach adenocarcinoma	1.59	0	0.23	0	0.23	0
Uterine corpus endometrial carcinoma	3.59	1.13	0	0.19	0	0
Bladder urothelial carcinoma	1	0	0	0	0	0
Esophageal adenocarcinoma	1.1	0	0	0	0	0
Skin cutaneous melanoma	1.35	0.22	0	0	0.22	0
Lung adenocarcinoma	1.06	0	0	0	0	0
Cervical squamous cell carcinoma	0.67	0.34	0	0	0	0
Head and neck squamous cell carcinoma	0.96	0	0	0.19	0	0
Lung squamous cell carcinoma	0.41	0	0	0	0.2	0
Pancreatic adenocarcinoma	1.09	0	0	0	0	0
Kidney renal papillary cell carcinoma	1.06	0	0	0	0	0
Acute myeloid leukemia	0.5	0	0	0	0	0
Liver hepatocellular carcinoma	0.54	0	0	0	0	0
Glioblastoma multiforme	0.34	0	0	0	0	0
Brain lower grade glioma	0.2	0	0	0	0	0

## 6 Functions and Regulatory Mechanisms of Serine/Arginine-Rich Splicing Factor 6 in Tumorigenesis

**FIGURE 2:**
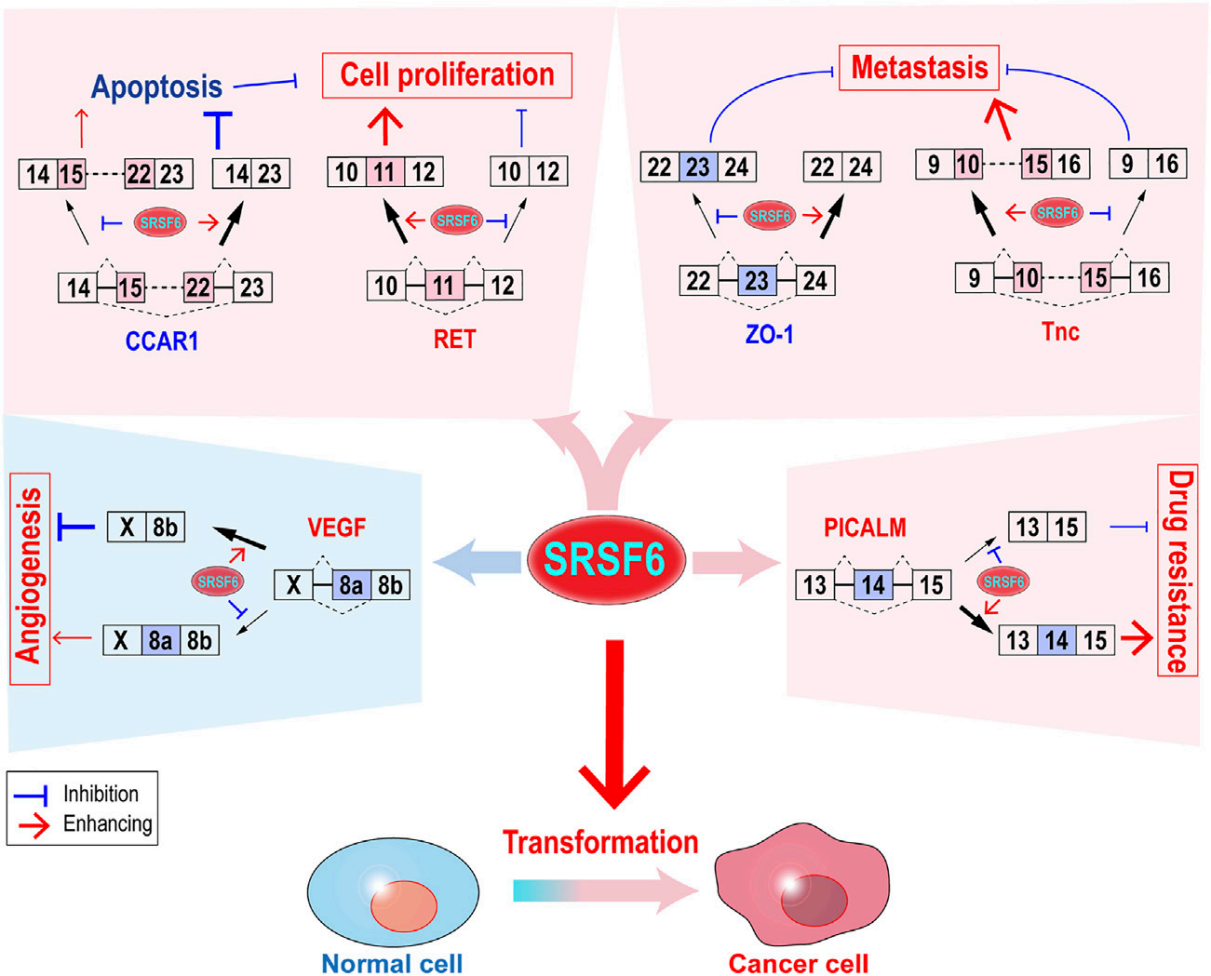
Functions and regulatory mechanisms of SRSF6 in tumorigenesis. SRSF6 can transform cells and control the alternative splicing of several target genes to promote cell proliferation, metastasis, and drug resistance. SRSF6 may also play anti-cancer function by inhibiting angiogenesis.

As a splicing factor, SRSF6 controls alternative splicing of a number of target genes, through which SRSF6 regulates almost all key aspects of tumorigenesis (Figure 2), such as transformation, cell proliferation, metastasis, immunosuppression, and drug resistance.

### 6.1 Transformation

The overexpression of some SR proteins, such as SRSF1 (Karni et al., 2007; Anczukow et al., 2012) or SRSF3 (Jia et al., 2010) can induce cell transformation. It is also true for SRSF6. Similar to SRSF1 and SRSF3, SRSF6 overexpression also transforms mouse embryonic fibroblast (Jensen et al., 2014). In non-transformed mouse or human lung epithelial cells, SRSF6 overexpression enables these cells to form colonies in soft agar and tumors in nude mice (Cohen-Eliav et al., 2013). In addition, in non-transformed mammary epithelial cells, SRSF6 overexpression induced significantly larger and dysmorphic acini morphology and increased the amount of proliferating acini in a short period of time, thereby indicating the potent transformation capability of SRSF6 (Park et al., 2019). Notably, most breast cancers originate from the mammary acini, which is the basic anatomical unit of the mammary gland. SRSF6 regulates the alternative splicing of genes associated with cell-cycle regulation, apoptosis, and cell adhesion, specifically increases exon10 inclusion of ARAP2, which is essential for cellular focal adhesion formation (Park et al., 2019).

Although SRSF6 overexpression can transform cells, it cannot be ruled out that the depletion of SRSF6 may also be involved in tumorigenesis in some tissues. Splicing factor SRSF3 is an example of this. SRSF3 overexpression in mouse embryonic fibroblast induced significantly tumor formation (Jia et al., 2010). However, specific knockout of SRSF3 in mouse hepatocytes impaired hepatocyte maturation and metabolism and induced spontaneous hepatocellular carcinoma (Sen et al., 2015). SRSF3 is required for protecting mice from tetrachloride-induced fibrosis and carcinogenesis in liver (Sen et al., 2015). Later, in human hepatocellular carcinoma cells, SRSF3 was found to be dephosphorylated and inactivated (Chen et al., 2021a). Therefore, the precise function of SRSF6 in a specific type of tissue or cell should be evaluated individually. In general, SRSF6 overexpression may be positively associated with cancers.

### 6.2 Cell Proliferation

#### 6.2.1 SRSF6 is Required for Cancer Cell Proliferation

Unlimited cell proliferation is the key characteristic of cancer. Knockdown or the use of specific inhibitor of SRSF6 significantly inhibited colorectal cancer cell proliferation (Wan et al., 2019). SRSF6 silence also significantly inhibited colon cancer and lung cancer cell proliferation, colony formation in soft agar, and eventually repressed tumor formation (Cohen-Eliav et al., 2013; Kim et al., 2016). By contrast, SRSF6 overexpression significantly enhanced the proliferation of immortal mouse lung epithelial cells (Cohen-Eliav et al., 2013). SRSF6 is also essential for T-cell acute lymphoblastic leukemia (T-ALL) cell proliferation and cell cycle progression (Zhou et al., 2020b). Moreover, inducible SRSF6 overexpression in transgenic mouse caused severe hyperplasia in mouse skin, as well as in the intestine, characterized by high level of cell proliferation and failure of epithelial cell differentiation and maturation (Jensen et al., 2014). SRSF6 seems to promote the initial steps of differentiation, but inhibit terminal differentiation in epithelial cells (Jensen et al., 2014). SRSF6 can also promote normal cell growth. Fernando et al. found that the overexpression of SRSF6 induced strong upregulated transcriptional level of oncogene Myc and enhanced cell growth in Drosophila (Fernando et al., 2015).

#### 6.2.2 Serine/Arginine-Rich Splicing Factor 6 Promotes Cell Proliferation via Multiple Molecular Mechanisms

SRSF6 promotes cancer cell proliferation via several molecular mechanisms, including repressing apoptosis, modifying energy metabolisms, and activating oncogenic signal transduction.

Cancer cells proliferate faster and undergo less apoptosis than normal cells. Cell cycle and apoptosis regulator 1 (CCAR1) gene is a transcriptional coactivator in apoptosis signaling pathway (Ou et al., 2009). CCAR1 has two isoforms produced by alternative splicing of exon 15–22. Full-length isoform encodes the pro-apoptosis CCAR1 protein. By contrast, isoform without exon 15–22 encodes an anti-apoptosis truncated CCAR1 protein. In T-cell acute lymphoblastic leukemia, SRSF6 inhibits apoptosis by binding to 3’ splice site of CCAR1 exon 22 and promoting exon 15–22 skipping (Lu et al., 2013). Moreover, a short isoform of Bim gene without exon 3 and 4, which is called BimS, is a potent apoptosis inducer. The overexpression of SRSF6 significantly reduced BimS isoform, and zinc ion can block SRSF6 binding to Bim RNA and induce cancer cell apoptosis by stimulating SRSF6 hyper-phosphorylation in normal HEK 293 cells (Hara et al., 2013). Another recent study showed that SRSF6 can increase the inclusion of Bcl-x exon 2b and produce more anti-apoptotic isoform Bcl-xL protein in 293T cells (Choi et al., 2021). However, SRSF6 overexpression might induce apoptosis by increasing BimS in melanoma cell line (Jiang et al., 2010; Lai et al., 2012). This phenomenon may be due to the higher expression levels of SRSF6 in cancer cells than in normal cells. Further increase in SRSF6 may cause some toxic effects in cancer cells.

SRSF6 can regulate energy metabolism to promote cell proliferation. Pyruvate kinase PKM gene has two isoforms, namely, M1 and M2, by mutual alternative splicing of exons 9 and 10 (Noguchi et al., 1986). M2 is mainly expressed in embryonic and cancer tissues and promotes cell proliferation (Christofk et al., 2008). SRSF6 overexpression specifically drives the splicing switch from M1 to M2 isoform (Jensen et al., 2014).

SRSF6 is responsible for the enhanced oncogenic signal transduction of oncogene RET. In medullary thyroid carcinoma (MTC), a somatic missense substitution mutation in the exon 11 of oncogene RET gene significantly increased the interaction between SRSF6 protein and exon 11 of RET RNA, and in turn increased the transcripts with the inclusion of exon 11 and the expression of full RET protein (Pecce et al., 2018), which may promote cell proliferation and tumorigenesis.

However, SRSF6 can also induce some tumor suppressive alternative splicing events. For example, FGFR1 has two isoforms generated by the alternative splicing of exon 3. The exclusion of exon 3 produces isoform FGFR1β, which is the preferred isoform in cancer and shows higher affinity for FGF1 than isoform FGFR1α in bladder cancer cells (Tomlinson and Knowles, 2010). The knockdown of SRSF6 significantly induced the switch from FGFR1α to FGFR1β (Jin and Cote, 2004). Unsurprisingly, SRSF6 shows some anti-tumor effects, which may be overwhelmed by its oncogenic effects in most cancer cells.

### 6.3 Metastasis

SRSF6 can promote cancer cell metastasis. SRSF6 overexpression increased migration and invasion in breast cancer cells (Park et al., 2019) and induced epithelial–mesenchymal transition (EMT) in colorectal cancer cells (Kong et al., 2016). Knockdown or using specific inhibitor of SRSF6 significantly inhibited colorectal cancer cell migration and invasion *in vitro* and metastasis *in vivo* (Wan et al., 2019). In principle, SRSF6 silence increased exon 23 inclusion of ZO-1 gene, which is an important cell adhesion molecule. ZO-1 isoform with exon 23, not isoform without exon 23, showed significant inhibitory role in cell motility (Wan et al., 2019). In skin cancer, SRSF6 promoted exon 10-15 inclusion of Tnc (extracellular-matrix protein tenascin C) gene, which can promote cell migration through its isoform with 10–15 exons (Jensen et al., 2014).

### 6.4 Immunosuppression

Immunosuppression helps cancer cells to escape from the immune system and progress. PBMCs in breast cancer patients contains several immune suppressive cells, such as myeloid-derived suppressor cells (MDSCs). Interestingly, peripheral mononuclear cells (PBMCs) from patients with metastasis showed dramatic increase in SRSF6 RNA than those without metastasis (Moradpoor et al., 2020). So far, little is known about the function of SRSF6 in cancer immunosuppression.

SRSF6 regulates the expression of a set of immune-associated genes. For example, IL-1b, the first altered signal was induced by SRSF6 (Jensen et al., 2014) upon wounding (Morasso and Tomic-Canic, 2005). CD44, a cell surface adhesion molecule, mediates T-cell homing (de la Hera et al., 1989), as well as tumor metastasis (Chen et al., 2018). The knockdown of SRSF6 increased CD44 alternative exon v7 and v10 inclusion in U2OS cells (Filippov et al., 2007). However, SRSF6 overexpression decreased exon v6 inclusion in breast cancer cells (Loh et al., 2016). CD45 is a transmembrane tyrosine phosphatase expressed by all leucocytes (Charbonneau et al., 1988) and required for TCR-mediated T cell activation (Pingel and Thomas, 1989). During T cell activation, the expression level of SRSF6 significantly increases, thereby promoting CD45 exon 4 inclusion (Lemaire et al., 1999). SRSF6 may regulate cancer-associated immunosuppression via these genes. In addition, Lu et al. found that the genome-wide modification of pre-mRNA alternative splicing induced neoantigens and elicited anti-tumor immunity (Lu et al., 2021), which showed a new way to enhance cancer immunotherapy. The suppression of SRSF6 expression or function may also induce neoantigens for immunotherapy because SRSF6 controls a number of target genes.

### 6.5 Drug Resistance

SRSF6 overexpression can increase the resistance of immortal mouse lung epithelial cells to cis-platinum treatment (Cohen-Eliav et al., 2013). In gastric cancer, SRSF6 is required for the resistance of gastric cancer cells to oxaliplatin and 5-FU. In principle, SRSF6 promotes phosphatidylinositol-binding clathrin assembly protein (PICALM) exon 14 inclusion to produce a full-length PICALM protein, which is required for the autophagy-induced resistance of gastric cancer cells to oxaliplatin and 5-FU (Zhang et al., 2021). By contrast, PICALM protein without exon 14 sensitizes cancer cells to chemotherapy (Zhang et al., 2021). PICALM participates autophagic precursor formation (Moreau et al., 2014) and can form a PICALM-MLLT10 fusion gene in leukemia, which is often associated with poor outcome (Savage et al., 2010).

### 6.6 Angiogenesis

SRSF6 controls the alternative splicing of many target genes. Sometimes, SRSF6 may play an anti-tumorigenesis role. Angiogenesis is an example of this. VEGF is a key regulator of angiogenesis in cancers. The alternative splice acceptor site usage in exon 8 (a terminal exon) produce two isoform families of VEGF. Isoforms that use proximal acceptor site encode pro-angiogenic VEGFxxx proteins. On the contrary, isoforms that use distal acceptor site encode anti-angiogenic VEGFxxxb proteins (Bates et al., 2002). SRSF6 binds to a 35-nucleotide motif in exon 8 and promotes the usage of distal acceptor site and VEGF165b expression (Nowak et al., 2008), which was further confirmed in systemic sclerosis patients (Manetti et al., 2011). Therefore, in terms of angiogenesis, increased SRSF6 may inhibit angiogenesis and have an adverse effect on tumor development.

However, VEGF165b isoform expression actually decreased in colorectal cancer (Diaz et al., 2008), which had SRSF6 overexpression. Other splicing factors may function against SRSF6 to suppress VEGF165b expression in cancers. Nowak et al. showed that oncogene SRSF1 can inhibit VEGF165b expression by relatively suppressing distal acceptor site usage (Nowak et al., 2008).

### 6.7 Serine/Arginine-Rich Splicing Factor 6 and Wound-Healing

Cancer is considered a kind of aberrant wound healing process (Dvorak 1986; Sundaram et al., 2018). For example, skin cancer shares similar gene-expression profile with wounded normal skin (Schafer and Werner, 2008). Notably, SRSF6 overexpression dramatically upregulated the expression of a set of genes involved in wound-healing by 13–154 fold, including keratin 6, keratin 16, IL-1b, Cxcl2, and Ccl3 in a transgenic mouse model (Jensen et al., 2014). Normal skin may only upregulate SRSF6 expression for several days after injury in contrast to the continuous SRSF6 overexpression in cancer (Jensen et al., 2014). This study revealed that SRSF6 exerted an important role in wound healing process, as well as in cancer when continuously overexpressed.

## 7 Regulatory Mechanisms of Serine/Arginine-Rich Splicing Factor 6 Expression and Function

**FIGURE 3:**
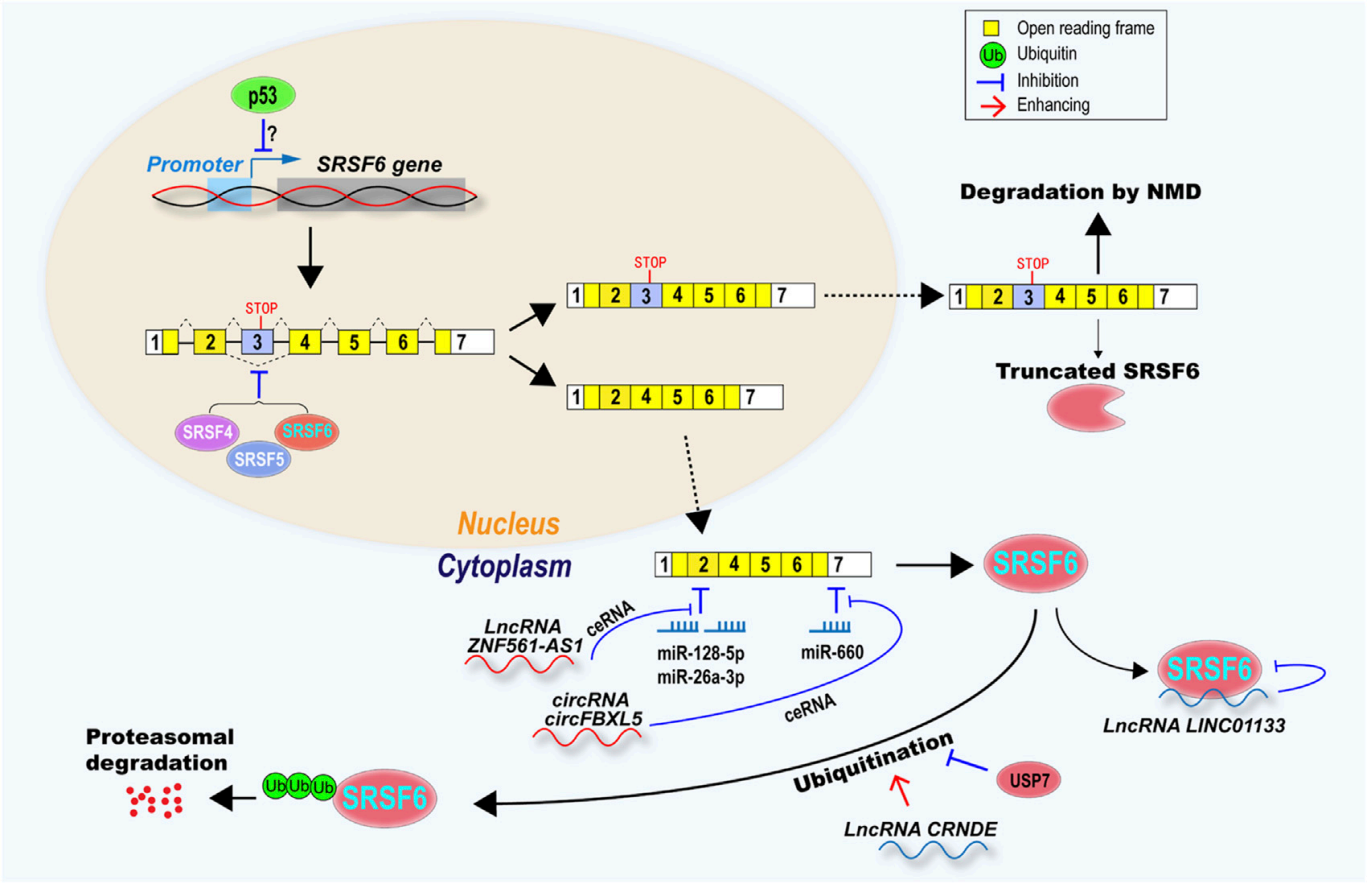
SRSF6 expression and function were regulated at multiple levels, including transcription, alternative splicing, mRNA stability, protein stability and function. Tumor suppressor p53 may inhibit SRSF6 transcription. SRSF6 itself, SRSF4, and SRSF5 inhibit SRSF6 exon 3 exclusion to produce a transcript with exon 3, which is a target of NMD or encode a truncated SRSF6 protein. Transcripts without exon 3 encode full-length SRSF6, and were inhibited by microRNA miR-128-5p, miR-26a-3p, and miR-66a, but rescued by lncRNA ZNF561-AS1, circRNA circFBXL5 via ceRNA mechanism. SRSF6 protein can be suppressed by lncRNA LINC01133. SRSF6 can be degraded via ubiquitination-proteasome. LncRNA CRNDE promotes SRSF6 ubiquitination, however, USP7 deubiquitinase can protect SRSF6 from ubiquitination and degradation.

Splicing factors often control a number of alternative splicing events in cells, which have tremendous effects on multiple cellular biological processes. Therefore, to tightly maintain the relative stable SRSF6 expression level, cells have developed various regulatory pathways at multiple levels, including transcription, splicing, translation, protein stability, and function (Figure 3).

### 7.1 Alternative Splicing of Serine/Arginine-Rich Splicing Factor 6 Poison Exon 3

An important regulatory mechanism of SRSF6 expression is the inclusion of its alternative exon 3, which is conserved across multiple species (Lareau and Brenner, 2015). Transcripts including this poison exon, which contains a pre-mature stop codon, are mostly degraded by nonsense-mediated decay (NMD) and may also encode a truncated SRSF6 protein. The inclusion of exon 3 is positively regulated by Nova1 (Lin et al., 2016), SRSF4 and SRSF5 (Leclair et al., 2020), and leads to reduce full-length SRSF6 protein level. SRSF6 can also promote the inclusion of exon 3 and autoregulate its own expression, which is also a conserved autoregulatory mechanism in SR family (Leclair et al., 2020). This mechanism exists not only in carcinoma cells, but also in leukemia cells (Leclair et al., 2020; Zhou et al., 2020b). Cancer cells prefer to impair autoregulatory mechanism and produce transcripts without exon 3 and then increase SRSF6 protein level (Zhou et al., 2020b). This phenomenon also exists in other SR proteins. For example, SRSF3 poison exon 4 inclusion is significantly downregulated in oral cancer (Guo et al., 2015).

### 7.2 Competing Endogenous RNAs

Competing endogenous RNA (ceRNA) regulation is another important regulatory mechanism of SRSF6 expression. CeRNAs can attenuate the inhibition of SRSF6 expression mediated by microRNAs. SRSF6 is targeted by miR-660. circFBXL5 functions as a ceRNA to sponge miR-660 and upregulate SRSF6 expression in breast cancer (Zhou et al., 2020a). Another example is that lncRNA ZNF561-AS1 can sponge miR-26a-3p and miR-128-5p to upregulate SRSF6 expression in colorectal cancer (Si et al., 2021).

### 7.3 Ubiquitination

Ubiquitination is an important regulatory mechanism of protein stability mediated by the ubiquitin-proteasome system (Hershko, 1983). Several splicing factors have been found to be ubiquitinated and then degraded by proteasome (Du et al., 2021). LncRNA can directly regulate SRSF6 protein stability or function. For example, lncRNA CRNDE binds to SRSF6 protein and causes its ubiquitination and degradation by proteasome in gastric cancer cell (Zhang et al., 2021). In T-ALL, SRSF6 expression was enhanced by the increased USP7, an ubiquitinase, which can deubiquitinate and stabilize SRSF6 protein (Zhou et al., 2020b).

### 7.4 Antagonistic Protein

The oncogenic function of SRSF6 may be neutralized by its antagonistic protein. For example, by using a genetic screen of randomly overexpressing genes, Fernando et al. discovered that the brat (brain tumor protein) gene of Drosophila, a tumor suppressor and post-transcriptional repressor of myc, can overcome the effects caused by SRSF6 overexpression (Fernando et al., 2015). The human homolog of brat is TRIM3 gene, which also suppresses tumorigenesis by ensuring asymmetric cell division of neural cells, attenuating stem-like characteristics of glioblastoma cells, and suppressing c-Myc expression (Chen et al., 2014).

### 7.5 Other Regulators

SRSF6 expression is also regulated by a list of key tumor-related genes. For example, DNA damage can induce SRSF6 expression in colorectal cancer cells lacking p53, not in cells with p53 expression, indicating that p53 may downregulate SRSF6 in cancer cells (Filippov et al., 2007). On the other hand, SRSF6 may also regulate alternative splicing of p53 pre-mRNA, which often mis-spliced due to mutations in SRSF6 binding motifs in Li–Fraumeni and Li–Fraumeni-Like syndrome patients, two hereditary cancer predisposition syndromes commonly with somatic mutation in p53 (Kouidou et al., 2009). Estrogen indirectly inhibits SRSF6 expression in breast cancer cells. Estrogen receptor-positive (ER^+^) breast tumors had decreased abundance of SRSF6 compared with ER^−^ tumors (Lal et al., 2013). In colorectal cancer, lncRNA LINC01133 can block SRSF6 function in metastasis by interacting with SRSF6 protein. However, TGF-β can induce LINC01133 downregulation, and then allow SRSF6 to promote tumorigenesis in colorectal cancer (Kong et al., 2016). Pnn is a desmosome associated protein, and is overexpressed and associated with poor prognosis in cancers (Mini et al., 2019). Silence of Pnn significantly reduced SRSF6 expression (Chiu and Ouyang, 2006).

## 8 Current Methods for Targeting Serine/Arginine-Rich Splicing Factor 6 Expression and Function

**FIGURE 4:**
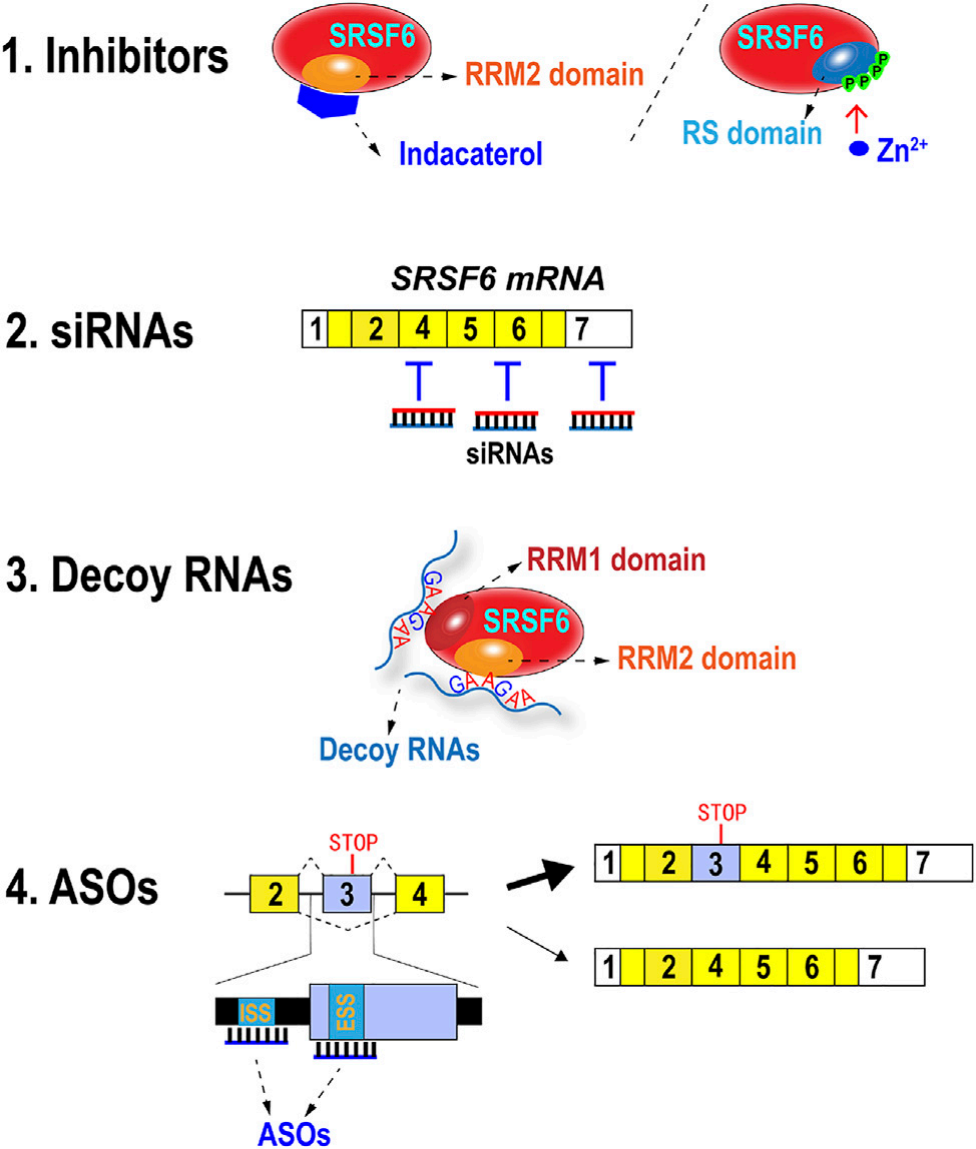
Methods of inhibiting SRSF6 expression and functions. Indacaterol suppresses SRSF6 by binding to RRM2 domain. Zn^2+^ can induce hyperphosphorylation of RS domain and abolish its RNA binding ability. SRSF6 mRNAs can be targeted and degraded by siRNAs. Decoy RNAs with SRSF6 binding motifs may block SRSF6 function. Antisense oligonucleotides targeting potential exonic splicing suppressor (ESS) or intron splicing suppressor (ISS) in or around exon 3 may promote exon 3 inclusion.

Apparently, SRSF6 functions as a potential oncogenic gene in numerous types of cancer. It may be an important target for cancer treatment. In fact, some strategies have been or can be developed to block SRSF6 expression or function for potential cancer therapy (Figure 4).

### 8.1 Inhibitors

Small molecules or metal ion are applied to inhibit SRSF6 function. Wan et al. found that stable knockdown of SRSF6 significantly decreased the xenograft tumor growth and lung metastasis in nude mice. Moreover, they predicted the 3D structure of SRSF6 protein RRM2 domain by simulation software and virtually screened possible chemicals that were able to bind the binding pockets of RRM2 domain (Wan et al., 2019). They discovered that indacaterol, a β2-agonist for the treatment of chronic obstructive pulmonary disease (COPD) (Yum et al., 2017), could significantly inhibit SRSF6 function, colorectal cancer cell proliferation, and tumor formation in a mouse colorectal model (Wan et al., 2019).These findings implied that RRM2 domains of other SR proteins may be promising targets of developing specific inhibitors because RRM2 domain is not conserved in SR proteins.

The hyperphosphorylation of SR proteins inhibited their splicing activity (Prasad et al., 1999). Hyperphosphorylation of SRSF6 also reduces its splicing activity and stability (Lai et al., 2003). Zinc ion markedly and specifically phosphorylated SRSF6 and induced its hyperphosphorylation and loss of RNA-binding ability (Hara et al., 2013), thereby suggesting that zinc ion may be applied to suppress SRSF6 function as an inhibitor.

### 8.2 Small Interfering RNAs

Small interfering RNAs (siRNAs) are synthetic double-stranded RNA and can interfere with its target gene expression. Anti-SRSF6 siRNAs can efficiently silence SRSF6 expression and may inhibit cancer cell proliferation and migration. However, the clinical application of siRNA is still facing many obstacles, such as off-target effects and *in vivo* delivery (Rautela et al., 2021). Therefore, the anti-tumor efficiency of anti-SRSF6 siRNA should be tested and improved *in vivo*.

### 8.3 Decoy RNAs

Decoy RNA oligonucleotide is another type of synthetic small RNA, which specifically binds to RNA binding proteins and blocks their function by steric hindrance (Denichenko et al., 2019). This strategy has been successfully used to target SR protein SRSF1 and three other splicing factors, namely, PTBP1 and RBFOX1/2. The splicing function of PTBP1 was interfered by decoys. Breast cancer cells treated with PTBP1 decoy showed significantly retarded cell proliferation and reduced soft agar colony formation (Denichenko et al., 2019). SRSF6 recognizes specific motifs in RNA, especially purine-rich motifs (Nagel et al., 1998). For example, SRSF6 binds a consensus motif sequence of UGGAG in ZO-1 exon 23 (Wan et al., 2019), a sequence of UGCAGGA in Tnc exon 12 (Jensen et al., 2014), and a sequence of AGTAGA in HIV-1 pre-mRNA (Erkelenz et al., 2015). Alvelos et al. identified thousands of SRSF6 binding motifs in human pancreatic β-cells by integrating individual-nucleotide resolution UV-cross-linking, immunoprecipitation (iCLIP) and RNA sequencing. Importantly, they found that SRSF6 preferred to bind a purine-rich consensus motif that contains GAA triplets, and more contiguous GAA triplets were associated with stronger binding (Alvelos et al., 2021). These studies paved the way to design efficient decoys to block SRSF6 binding and correct aberrant alternative splicing in cancers.

### 8.4 Antisense Oligonucleotides

Another promising approach is to take advantage of SRSF6 autoregulation mechanism by increasing the inclusion of its exon 3 with antisense oligonucleotides (ASOs) and then relatively decreasing the short isoform without exon 3, which encodes full-length oncogenic SRSF6 protein. This strategy has been successfully applied in decreasing overexpressed SRSF3 and inhibiting cell proliferation in oral cancer cells (Guo et al., 2018). Theoretically, this strategy depends on the identification of exonic splicing suppressors (ESSs) or intronic splicing suppressors (ISSs), which are responsible for the exclusion of SRSF6 exon 3. Then specific ASOs can be designed according to ESS or ISS sequence. These ASOs can bind to these motifs and block the interaction with regulatory factors and release the suppressive effects on exon 3 inclusion. The off-target effects of anti-splicing suppressor ASOs may be much less than siRNAs because splicing suppressors are hardly conserved in genome. However, similar to siRNAs, *in vivo* delivery is also the major obstacle for clinical application of ASOs (Gheibi-Hayat and Jamialahmadi, 2020).

## 9 Conclusion and Remarks

In summary, alternative splicing regulator SRSF6 is overexpressed in many types of cancer and associated with poor prognosis in some cancers. Moreover, SRSF6 plays important roles in most of key aspects of tumorigenesis by controlling the alternative splicing of the key tumor-associated genes and can transform cells when overexpressed. Therefore, SRSF6 is an oncogene and promising target for cancer therapy. Some anti-SRSF6 methods have been or can be developed.

However, some challenges need to be overcome further. First, only a few studies analyzed the relationship between SRSF6 expression and disease progress and prognosis of cancer. The diagnosis and prognosis value of SRSF6 in cancers remain largely unknown. Second, the functions of SRSF6 in cancer immunosuppression is unknown. The roles of SRSF6 in cancer immunosuppression should be explored because emerging evidences have revealed the important roles of alternative splicing in cancer immunotherapy (Frankiw et al., 2019). Third, besides alternative splicing, SRSF6 also plays roles in transcription and translation. Understanding whether SRSF6 regulates tumorigenesis via these processes will be interesting. Fourth, SRSF6 may potentially inhibit angiogenesis. The inhibition of SRSF6 expression or function may enhance angiogenesis. Many anti-angiogenic methods (Al-Ostoot et al., 2021), such as targeting the VEGF signaling pathway (Van Cutsem et al., 2020) are also available. It may be worthwhile to try to use the combination of an anti-angiogenic treatment to enhance the effects of anti-SRSF6 cancer therapy. Finally, most of SR protein family members are associated with tumorigenesis, and it remains largely unclear whether the functions of these proteins in tumorigenesis are redundant, complementary or even competitive.

## Abbreviations

ASOs, antisense oligonucleotides; CCAR1, cell cycle and apoptosis regulator 1; CeRNA, competing endogenous RNA; COPD, chronic obstructive pulmonary disease; EMT, epithelial–mesenchymal transition; ESSs, exonic splicing suppressors; iCLIP, UV-cross-linking and immunoprecipitation; ISSs, intronic splicing suppressors; MTC, medullary thyroid carcinoma; NMD, nonsense-mediated decay; PBMCs, peripheral mononuclear cells; PICALM, phosphatidylinostol-binding clathrin assembly protein; RRM, RNA recognition motif; RS, arginine and serine dipeptides; SRSF6, serine/arginine-rich splicing factor 6; T-ALL, T-cell acute lymphoblastic leukemia cells; Tnc, extracellular-matrix protein tenascin C.
